# Environmental impact of open burning of polyester and cotton textile waste: a comparative analysis

**DOI:** 10.1007/s11356-026-37563-9

**Published:** 2026-03-08

**Authors:** Jeyran Bayramova, Steven Pires, Patricia A. Holden, William J. Sagues, Richard Venditti, Jesse S. Daystar

**Affiliations:** 1 Cotton Incorporated, Sustainability, 6399 Weston Parkway, Cary, NC 27513 USA; 2 Department of Forest Biomaterials, North Carolina State University, 2820 Faucette Dr., Raleigh, NC 27607 USA; 3 Bren School of Environmental Science and Management, University of California, Santa Barbara, CA 93106 USA; 4 Department of Biological & Agricultural Engineering, North Carolina State University, 3110 Faucette Dr., Raleigh, NC 27695 USA

**Keywords:** Open burning, Textile waste, Atmospheric pollutants, Public health risk, Environmental impact

## Abstract

Open burning of waste (OBW), particularly textile waste, is a growing but under-researched global issue with critical environmental and public health implications. This study examines the scale and impact of open burning of textile waste (OBTW), focusing on polyester and cotton materials commonly found in post-consumer and industrial waste streams. While high-income countries strictly regulate open burning, it remains prevalent in low- and middle-income countries due to limited waste infrastructure and poor waste management, contributing to severe air pollution and greenhouse gas (GHG) emissions. Our analysis estimates that between 10 and 20 Mt (medium 15 Mt) of textile waste is burned annually across 137 countries, with polyester burning alone contributing 8.3 to 24.6 Mt of fossil carbon dioxide (CO_2_) emissions. The South Asia region accounts for 37% of these emissions, highlighting regional disparities in waste impacts. Open burning also releases hazardous pollutants such as particulate matter (PM_2.5_, PM_10_), polycyclic aromatic hydrocarbons (PAHs), and toxic heavy metals (THMs), disproportionately affecting vulnerable populations. Although natural fibers like cotton emit less CO_2_, their combustion can still produce high levels of carbon monoxide (CO) under specific conditions. A sensitivity analysis shows that uncertainty in polyester emissions is driven mainly by the textile fraction in MSW and polyester emission factors, whereas uncertainty in cotton emissions is primarily influenced by the assumed cotton fiber share within the textile waste stream. This demonstrates that current emission inventories may misestimate impacts unless improved textile composition data and fiber-specific emission factors become available. This study underscores the urgent need for improved data, targeted research on pollutant behavior during textile combustion, and the development of waste management strategies to reduce the environmental and health burdens of open-burning practices.

**Supplementary Information:**

The online version contains supplementary material available at 10.1007/s11356-026-37563-9.

## Introduction

Every year, the textile industry generates around 92 Mt of waste with China and the USA contributing the largest quantities, 20 and 17 Mt respectively (Tang 2023). Only 15–20% of textile waste is collected for reuse and recycling, while the rest is landfilled, dumped, incinerated, or otherwise mismanaged, with all causing environmental and health concerns (Md Shamsuzzaman et al. 2025). Among waste disposal methods, open burning of waste (OBW) has emerged as a practice having severe and persistent issues. Feedstocks to OBW are complex, frequently including apparel, footwear, home textiles, post-manufacturing textile waste (e.g., cutting scraps, unused fabric bolts) and unsold goods. The scale of OBW is particularly large in low- and middle-income countries (LMIC), where around 93% of municipal solid waste (MSW) is burned or dumped in open environments, compared to just 2% in high-income countries (HIC) (Kaza et al. 2018). Best estimates indicate that 40–65% of MSW in developing countries is openly burned, yet the composition share of that burned stream, including textiles, remains poorly quantified (Pathak et al. 2024; Hoffer et al. 2020). While illegal in some countries (e.g., the USA, Australia, EU), OBW remains poorly regulated or entirely unregulated in many developing nations. It is a widespread practice that is difficult to regulate and quantify (Hoffer et al. 2020). Global analyses by Cook and Velis (2021) suggest that nearly a billion tons of mixed waste may be burned annually in uncontrolled conditions, contributing significantly to air pollution.

The emission profile of OBW is often underestimated due to a lack of accurate data on waste feedstocks, open-burning events, and their associated emission factors (Wiedinmyer et al. 2014). This gap impedes a comprehensive understanding of the scale and impact of textile waste burning in the context of the overall OBW. Incomplete combustion emits GHGs and hazardous air pollutants such as particulate matter (PM_2.5_ and PM_10_), dioxins, furans, toxic heavy metals (THMs), and polycyclic aromatic hydrocarbons (PAHs) (Cheng et al. 2020). Plastic waste, including polyethylene terephthalate (PET)–based products commonly found in synthetic textiles such as polyester, is a key contributor to these pollutants (Abdel-Shafy & Mansour 2018; Simoneit et al. 2005). The toxic compounds from burning plastics pose serious health risks, including respiratory and cardiovascular diseases, cancer, and damage to the nervous and reproductive systems. Overall, waste mismanagement is implicated in 400,000 to 1 million premature deaths annually, with plastics likely a major driver (Williams et al. 2019).

The world produces over 400 Mt of plastic each year, of which more than 40 Mt are synthetic textiles (OECD 2019). Globally, plastic leakage reached 60 Mt in (Wernet 2019), with apparel contributing 14% of the total; end-of-life synthetic apparel accounted for 81% of this leakage (Kounina et al. 2024). Much of this material flows to developing countries through secondhand clothing exported by the USA, UK, EU, and Canada (Banik 2020; Sonnenberg et al. 2022). Ghana in East Africa alone imports over 15 million secondhand garments each for a population of just 32 million people, with at least 40% deemed unusable and ending up in dumpsites and open-pit burning scenarios (Besser 2021; Ricketts 2024). Infrared testing revealed that nearly 90% of these garments are made of synthetic fibers such as polyester (Greenpeace Africa 2024), exacerbating the country’s already critical plastic pollution crisis. The environmental consequences of open burning extend far beyond Ghana; in countries such as Comoros, Chad, Rwanda, and the Solomon Islands, carbon dioxide (CO_2_) emissions from OBW are reported to be three to four times higher than their total national anthropogenic CO_2_ emissions. In Mali and Burundi, this disparity is even greater, reaching approximately five times the countries officially reported national anthropogenic carbon dioxide emissions (Williams et al. 2019).

Similar dynamics affect parts of South Asia and Latin America, where inflows of secondhand and manufacturing textile wastes often end up in open-burning pits. This issue is worsened by a lack of systematic waste collection, particularly in hot climates where accumulated waste can become a breeding ground for germs, prompting open burning as a rapid solution to minimize some health risks (Cook and Velis  2021). Energy poverty can compound this. Bharadwaj et al. (2025) report 13% of surveyed households in Nigeria use plastic garbage for cooking fuel, while in Chad, more than 60% of urban residents rely on plastic waste for energy. A recent global systems modeling report by Pew and ICF shows that open burning already accounts for the largest share of downstream emissions in the plastic system (43% in 2025), rising to 59% by 2040. According to the same report, under the business-as-usual scenario, emissions from open burning are projected to increase by ~160%, driven by persistent gaps in waste collection and rapidly growing plastic waste generation (The Pew Charitable Trust & ICF 2025). These dynamics are expected to accelerate as global plastic production—including synthetic fibers—is projected to double within the next 10–15 years, likely overwhelming already strained waste management systems (Williams et al. 2019). Fast fashion trends are expected to raise apparel consumption from 62 Mt in2015 to 102 Mt by 2030, with industry waste increasing from 92 to 148 Mt (Global Fashion Agenda and Boston Consulting Group 2017) . Consequently, post-consumer apparel waste will likely grow, further contributing to open-burning practices.

**Research gap and novelty**: Despite the scale of the problem, no prior research has quantified the global or regional burden of open burning of textile waste (OBTW) as distinct from general OBW. Existing inventories address MSW burning without isolating textile flows, while a few laboratory studies provide emission factors for a “textiles” category but do not quantify OBTW or differentiate between natural and synthetic fibers. This study is, to our knowledge, the first to estimate OBTW at global and regional scales, and the first to compare the relative contributions of polyester and cotton using waste-system data and fiber-specific emission assumptions. By addressing these gaps and outlining further research needs, the study underscores the urgent need for improved global textile waste management practices to mitigate environmental and health risks.

**Study scope**: We assessed global and regional estimates of OBW using waste management data from the World Bank’s dataset, which encompasses residential, commercial, and institutional waste while excluding industrial, medical, hazardous, and construction waste. As the dataset covers the period 2011–2017, some critical countries where OBW scenarios are common practices were updated with more recent literature.

## Materials and methods

**Fig. 1:**
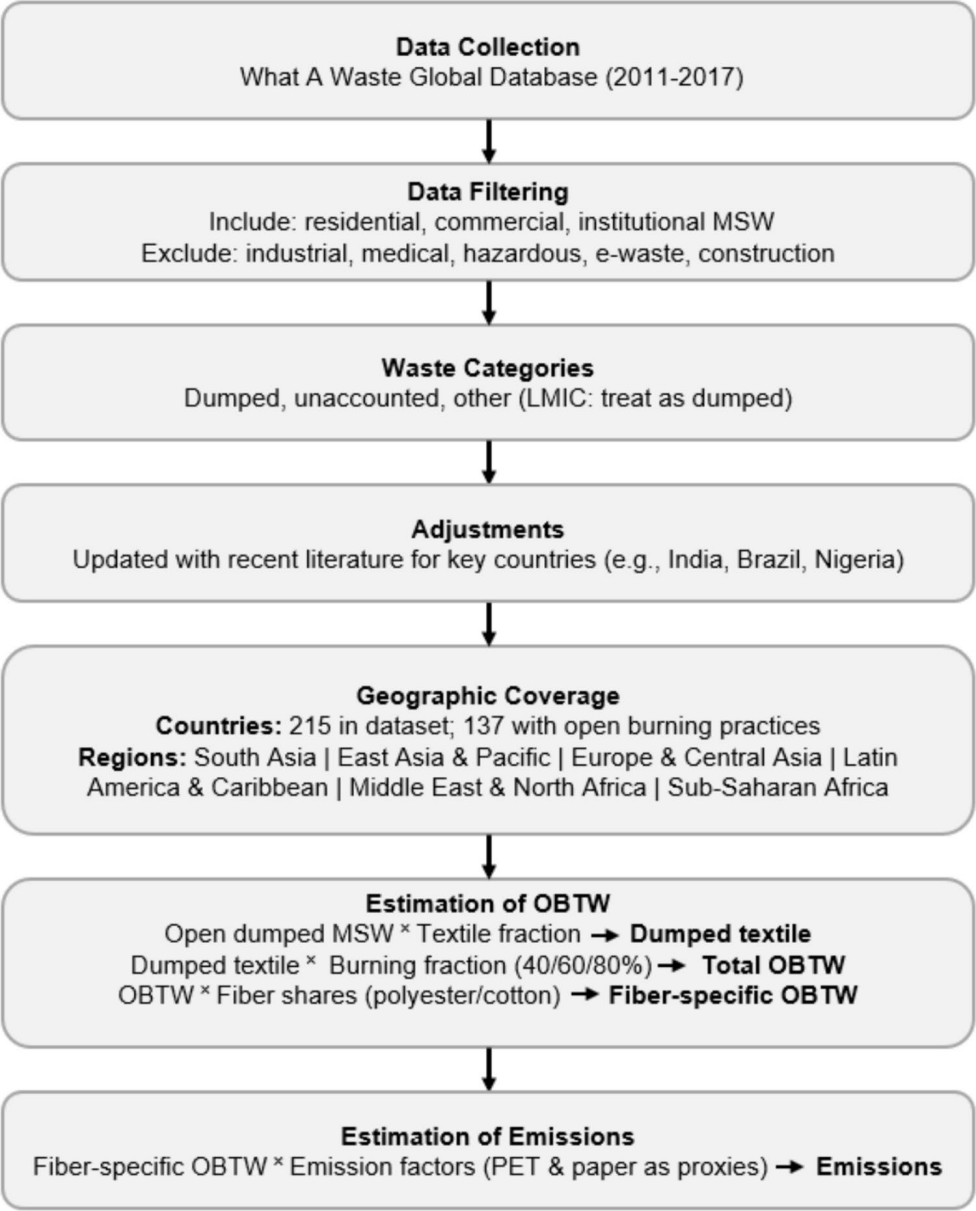
Workflow for estimating OBTW and associated emissions

To provide a clear overview of the methodological approach, the full data-processing and estimation workflow is presented in Fig. 1 before the detailed description of each step. The figure outlines the sequence from MSW data collection and filtering to the estimation of fiber-specific textile waste burning (i.e. OBTW) and associated emissions.

### Waste quantities and country coverage

The waste quantities were derived from each country’s total MSW generated annually, and the proportion classified as dumped. The dataset originates from the World Bank’s What A Waste Global Database (World Bank 2024), which reports MSW data for individual countries at varying years between 2011 and 2017. For countries in regions with the greatest extent of open dumping (e.g., India, Brazil, Nigeria), values were updated using more recent literature to improve accuracy for high-impact cases, whereas the World Bank’s dataset remained the primary source for all other countries. Along with the dumped category of waste, we considered “unaccounted” and “other” categories of waste. It is assumed that waste which is not processed (“unaccounted” category) by formal disposal methods, such as landfilling or recycling, is either openly dumped or burned, whereas in low- and middle-income countries, waste classified in the category of “other” is also presumed to be dumped (Kaza et al. 2018). The fate of dumped waste is frequently linked with open burning, particularly in LIC where infrastructure is limited. UNEP (2025) stated that discarded clothing often flows to regions with insufficient waste management, where OBW remains a common practice. On this basis, we treat open dumped waste as being a high risk of on-site burning. Out of 215 countries listed in the World Bank’s waste management data, 137 fall under the openly dumped waste criteria, including “other” and “unaccounted” waste. These countries were grouped into six regions: South Asia, East Asia & Pacific, Europe & Central Asia, Latin America & Caribbean, Middle East & North Africa, and Sub-Saharan Africa. High-income countries, such as the USA, Canada, Australia, and most of the EU states are excluded from this list, as open dumping and open burning are strictly prohibited in these nations; consequently, no such data is reported in the World Bank’s dataset.

### Textile waste estimation

To estimate the amount of textile waste burned, we applied each country’s textile waste fraction, based on either available data or a global average (3%), to the total volume of dumped waste (Table 1). This approach assumes that the composition of dumped waste reflects that of the total MSW, with the proportion of textiles being consistent across both.

**Table 1:** Textile fraction in MSW composition

Country	Textile waste fraction Textile mass/total mass × 100	Reference
India	4%	Awasthi et al. (2023)
Afghanistan	1%	UNEP (2017)
Malaysia	3%	Marippan et al. (2023)
Bangladesh	3%	Waste Concern (2021)
Brunei	2%	UNEP (2017)
Cambodia	3%	Pheakdey et al. (2022)
China	3%	Zhu et al. (2020)
Indonesia	3%	Qonitan et al. (2021)
Myanmar	2%	UNEP (2017)
Nepal	3%	Khatoon (2020)
Pakistan	2%	ITA (2024)
Philippines	2%	UNEP (2017)
Republic of Korea	2%	UNEP (2017)
Singapore	2%	UNEP (2017)
Uganda	3%	Wasteaid (2024)
Tanzania	2%	Singh (2021)
Mexico	5%	Galicia et al. (2019)
Brazil	6%	Lino et al. (2023)
Thailand	5%	Eaktasang et al. (2019)
Vietnam	2%	Awasthi et al. (2023)
Ghana	3%	Seshie et al. (2020)
Chile	7%	Pérez et al. (2022)
**Global average**	**3%**	Weighted global textile fraction

We assume that textile waste ending up in dumpsites or residential backyards is subject to burning as part of routine waste management practices. However, recognizing that not all dumped waste gets burned, we followed the IPCC’s guidance, which estimates that 60% of open waste under burning scenarios is actually burned (IPCC 2006). Gómez-Sanabria et al. (2022) noted that this default may be inadequate in some regions, with lower fractions observed in low-income rural settings dominated by organic waste, and higher fractions where uncollected waste is more frequently burned. To capture this variability, we therefore applied a sensitivity range of 40–80%, with 60% as the central estimate. For further specificity, the fractions of polyester and cotton within textile waste were derived from the most recent data from the Textile Exchange Materials Market Report (Textile Exchange 2024), which reveals global production percentages for these fibers.

- **Polyester textile waste**: Polyester is the most widely produced synthetic fiber globally. The fraction of overall textile waste that is polyester was estimated by taking the global fraction of textile production that is polyester (57%) and subtracting the portion that is of recycled polyester textile (~1%), resulting in a 56% fraction of total textile waste that is polyester.
- **Cotton textile waste**: Cotton is the leading natural fiber in textile production. The cotton textile fraction of overall textile waste was estimated by taking the fraction of textile production that is cotton (20%) and subtracting the percentage that is of recycled cotton textile (1%), resulting in a 19% fraction of textile waste that is cotton.

These fractions were multiplied by the textile waste mass in each country to estimate the contribution of both polyester and cotton to the total volume of waste being burned (IPCC 2006).

The amount of waste burned is determined by the following formula and assumptions:

$${W}_{b}={W}_{d}\times {B}_{fraction}$$

where

- *W*_*b*_ = the amount of waste burned
- *W*_*d*_ = the amount of dumped waste (including “unaccounted” and “other” categories of waste)
- *B*_*fraction*_ = the burning fraction, with values of 40% (low), 60% (the default IPCC value), and 80% (high).

### Emissions to air

Air emissions from openly burned textile waste were estimated by multiplying the volume of OBTW by pollutant-specific emission factors derived from four different studies. Due to the limited availability of direct emission data for synthetic (PET) and natural textile waste (cotton), we used factors from complementary sources, including laboratory combustion experiments, field-based observations, and standardized databases. These studies were selected because they provide pollutant-specific data under different burning conditions and cover both synthetic and cellulose-based materials. For synthetic fibers, particularly polyester, emissions were considered analogous to those from plastic burning (e.g. PET, PE). In the case of cotton textiles, emissions were assumed to resemble those from paper combustion, as both cotton (82–96%) and paper (90–99%) are predominantly composed of cellulose (Ansell & Mwaikambo 2009; Sahin & Arslan 2008). The use of multiple datasets enables representation of combustion variability typical of open-burning conditions, including differences in combustion efficiency, oxygen availability, and burning phase. The combustion contexts and data sources are summarized in Table 2, while emission factors applied are reported in Table 3.

**Table 2:** Source studies and combustion contexts used in emission modeling

Source	Burning context	Proxy material	Pollutants covered	Model application
Cheng et al. (2020)	Outdoor OBW (barrel and pile)	Plastic and paper	CO_2_, CO, SO_2_, NOx, PM_2.5_, THMs	Characterizes uncontrolled field burning phases (flaming, transition, smoldering)
Wernet et al. (2016) (ecoinvent v3.5 database)	Standardized open burning	PE and graphical paper (paper waste stream)	CO_2_, CO, CO_4_, SO_2_, NOx, PM_2.5_, PM_10_ PAHs, THMs	Baseline emission factors
Hoffer et al. (2020)	Household stove burning	PET and paper	PM₁₀, PAHs, PAHs(BaP)	Low-oxygen combustion conditions
Wang et al. (2023)	Laboratory-based combustion	PE and paper	CO_2_, CO, NOx, SO2, PM_2.5_, PM_10_	Simulated open-burning conditions

Plastic-based materials were used as proxies for polyester textiles and paper-based materials as proxies for cotton due to the absence of textile-specific emission factors under open-burning conditions

**Table 3:** Emission factor dataset from different studies on OBW

Fuel (waste)	Method applied	Region	Emission factor, g kg^−1^												
			Fossil CO_2_^a^	Biogenic CO_2_^b^	CO	NOx	SO_2_	CH_4_	PM_2.5_	PM_10_	PAHs	PAHs (BaP)^c^	THMs^d^ (As)	THMs (Pb)	THMs (Zn)
Plastic	Barrel (Cheng et al. 2020)	China	1.80E+03		4.34E+01	3.10E+00	5.00E−01		1.30E+01				1.90E+00	1.20E+00	3.80E−01
Plastic	Pile (Cheng et al. 2020)	China	1.00E+03		2.03E+01	1.10E+00	3.00E−01		4.50E+00				1.30E+00	1.10E+00	1.70E−01
Plastic	Field (ecoinvent v3.5 database)	Global	2.92E+03		3.86E+01	2.39E+00	1.84E−01	5.90E+00	9.85E−01	3.79E−01	3.44E−01		1.55E−05	1.90E−04	2.54E−03
Plastic	Winter stove (Hoffer et al. 2020)	Europe								1.10E+01	3.20E−02	2.20E−03			
Plastic )	Lab (Wang et al. 2023)	South Africa	2.93E+03		2.24E+01	1.50E+00	8.00E−02		3.40E+01	3.66E+01					
Paper	Barrel (Cheng et al. 2020)	China		1.40E+03	6.66E+01	3.30E+00	3.00E−01		1.05E+01				1.00E+00	1.90E+00	2.90E−01
Paper	Pile (Cheng et al. 2020)	China		1.00E+03	3.41E+01	2.30E+00	2.00E−01		6.00E+00				1.20E+00	2.00E+00	1.20E−01
Paper	Field (ecoinvent v3.5 database)	Global		1.40E+03	3.86E+01	5.38E+00	6.06E−01	5.90E+00	1.04E+00	4.02E−01	3.44E−01		1.75E−05	6.75E−04	9.29E−04
Paper	Winter stove (Hoffer et al. 2020)	Europe								2.20E+00	1.20E−03	1.60E−04			
Paper	Lab (Wang et al. 2023)	South Africa		1.50E+03	4.49E+01	1.14E+00	5.70E−01		1.33E+01	1.34E+01					

^a^Burning fossil-based products like plastics, which are made from oil, releases fossil carbon into the atmosphere. ^b^Biogenic carbon emissions come from biological sources such as plants, trees, and soil and are part of the natural carbon cycle. ^c^Emissions of total polycyclic aromatic hydrocarbons (PAHs) expressed in benzo(a)pyrene (BaP) toxicity equivalent. ^d^Toxic heavy metals (THMs) substances contained in PM_2.5_ emissions

### Sensitivity analysis

A one-at-a-time sensitivity analysis was performed to assess the influence of five parameters on CO_2_ emissions from OBTW: textile fraction in MSW (1–7%, baseline = 3%), share of textiles burned (40–80%, baseline = 60%), fiber share (polyester = 45–70%, baseline = 56%; cotton = 10–30%, baseline = 19%), fiber-specific emission factors for CO_2_ (Table 3), and industrial pre-consumer offcuts entering MSW (0–10%, baseline = 5%). Offcuts are defined here as pre-consumer textile scraps generated during garment and fabric manufacturing including cutting waste, trimmings, selvage edges, and rejected pieces, that are sometimes improperly mixed into municipal waste streams rather than recycled or formally collected. The range for the *industrial offcuts → MSW* parameter was anchored to case studies documenting indiscriminate disposal (Silas-Ufelle 2025), evidence of landfill and burning routes for cutting waste (Hoque et al. 2024), and national ecosystem mappings that reveal large pre-consumer volumes and incomplete segregation (Phan et al. 2024). Each parameter was trialed with low and high values drawn from literature ranges, while holding others constant at baseline. Results were visualized as tornado diagrams, showing percent deviation from baseline emissions. This approach highlights which parameters most strongly affect uncertainty in emission estimates.

## Results and discussion

### Dump waste estimates

Using World Bank waste management data, we estimate that open dumped waste, including “other” and “unaccounted” waste treatment categories, occurs in 137 countries across six regions: Europe & Central Asia, East Asia & Pacific, South Asia, Latin America & the Caribbean, Sub-Saharan Africa, and Middle East & North Africa. Collectively, these regions annually generate more than 700 Mt of dumped waste representing 37% of the ~2 billion tons of MSW produced (Kaza et al. 2018). These estimates exclude waste directly discharged into waterways or marine environments.

**Fig. 2:**
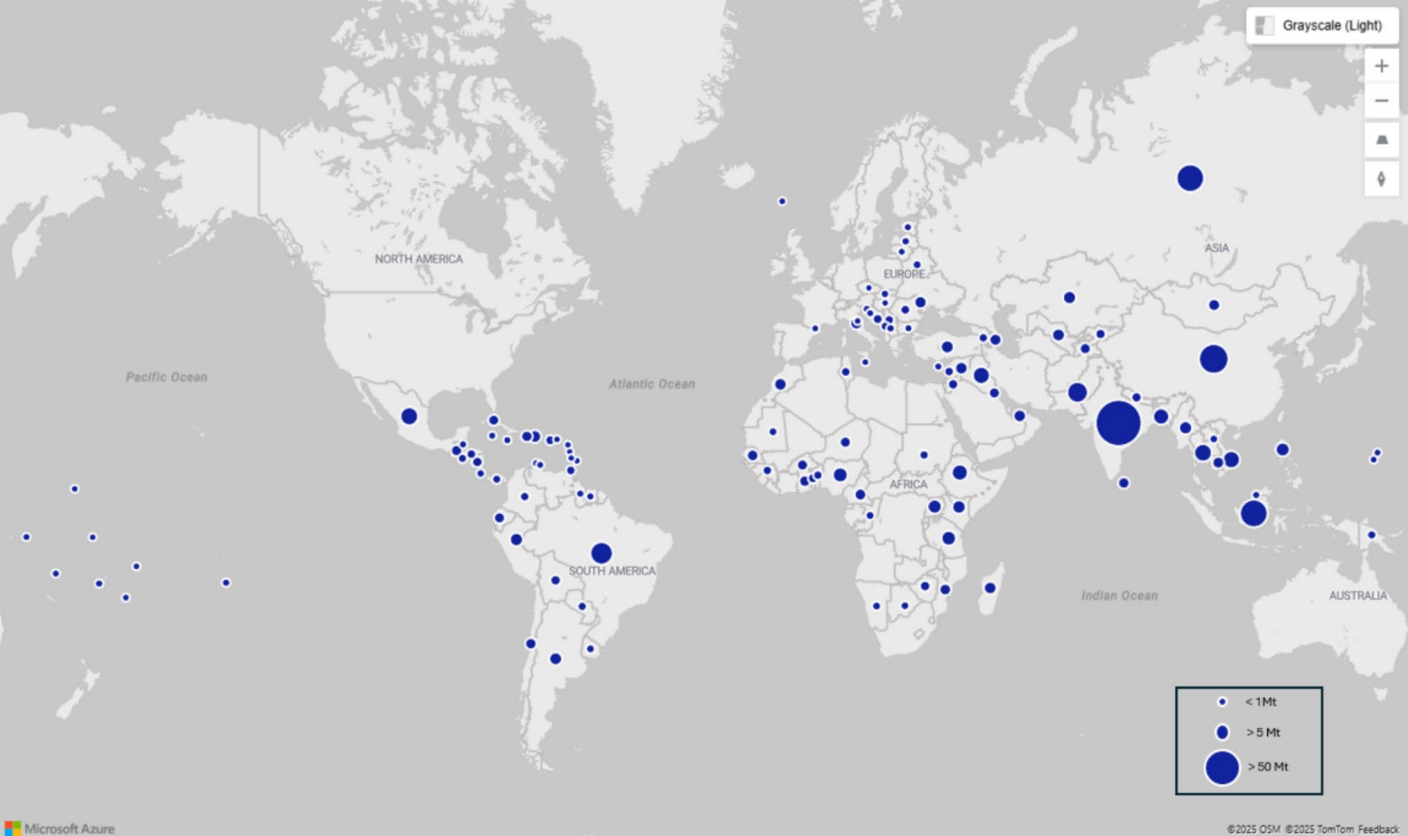
Global volumes of open dumped waste. Bubble size represents the volume of open dumped waste, with larger bubbles indicating higher waste volumes. The USA, Australia, and most EU countries were omitted as their proportions of open dumped waste are either not presented in the dataset or very negligible

Although comprehensive, these figures remain conservative, given limited collection and reporting in many low- and middle-income regions. Previous research suggests that nearly 40% of global MSW is openly burned. Our results confirm that South Asia and East Asia & Pacific dominate in volumes of dumped waste (Table 5) with India alone generating 213 Mt annually, accounting for over 70% of its MSW (Table 4). China follows with 70 Mt, though its incineration (controlled, high-temperature waste combustion) rates have risen sharply (to 40% by 2019), reducing uncontrolled disposal (WBG Report 2019). Other major contributors include Indonesia (58 Mt), Brazil (32 Mt), and Pakistan (25 Mt) (Table 4). The global distribution of open dumped waste is illustrated in Fig. 2, while Figure S1 presents waste generation by region and country, along with the proportion that is dumped.

**Table 4:** Annual country-level estimates of openly dumped MSW values, with per capita dumped waste and per capita OBTW (based on medium values), population and literature sources. Weighted averages for other countries and totals are included

Country	Open dump Mt	Open dump kg/per capita	OBTW kg/per capita	Population (2018)* Mn	Sourced literature for MSW and open dump
India	213.3	155	3.8	1,375	World Bank (2021)
China	70.0	50	0.9	1,403	WBG Report (2019)
Indonesia	58.1	215	3.9	270	Qonitan et al. (2021)
Brazil	31.9	155	5.2	206	Lino et al. (2023)
Pakistan	24.8	109	1.3	227	ITA (2024)
Egypt	22.7	215	1.5	106	Alsobky et al. (2023)
Mexico	15.6	125	3.8	125	Rueda-Avellaneda et al. (2021)
Thailand	14.9	208	5.9	71	Sukma et al. (2022)
Vietnam	13.5	140	1.7	96	Nguyen et al. (2024)
Ethiopia	10.7	95	1.7	113	Gebrekidan et al. (2024)
Bangladesh	10.5	64	1.2	164	Waste Concern (2021)
Chile	8.0	114	4.8	19	Cayumil et al. (2021)
Nigeria	8.0	39	0.8	205	Okafor et al. (2022)
Tanzania	7.4	128	1.5	57	Singh (2021)
Philippines	5.8	53	1.0	109	Premakumara et al. (2018)
Turkey	4.3	52	0.7	81	Melikoglu (2025)
Cambodia	2.7	167	3.0	16	Pheakdey et al. (2022)
Ghana	2.3	74	1.3	31	Kusi-Appiah et al. (2025)
Other countries (n = 119)	212	120*	2.1*	1,820	World Bank (2024)
All countries (n = 137)	736	113*	2.3*	6,493	World Bank (2024)

*Population values (2018) were used as a consistent reference year to ensure comparability across countries, as MSW data and literature updates originate from varying years. Values for “Oher countries” and “All countries” represent population-weighted averages. These were calculated using the population-weight sums of openly dumped MSW (kg/capita) and OBTW (kg/capita)

**Table 5:** Open dumped waste, textile waste dumped and burned, including synthetic (polyester) and cotton fibers measured in tons per year

Region	Range	Open dumped waste^a^	Textile waste dumped^b^	Textile waste burned^c^	Polyester burned^d^	Cotton burned^e^
South Asia	Low			3.87E+06	2.17E+06	7.35E+05
	Medium	2.52E+08	9.67E+06	5.80E+06	3.25E+06	1.10E+06
	High			7.74E+06	4.33E+06	1.47E+06
East Asia & Pacific	Low			2.18E+06	1.22E+06	4.14E+05
	Medium	1.74E+08	5.44E+06	3.26E+06	1.83E+06	6.20E+05
	High			4.35E+06	2.44E+06	8.27E+05
Europe & Central Asia	Low			9.80E+05	5.49E+05	1.86E+05
	Medium	8.87E+07	2.45E+06	1.47E+06	8.24E+05	2.79E+05
	High			1.96E+06	1.10E+06	3.73E+05
Latin America & Caribbean	Low			1.41E+06	7.90E+05	2.68E+05
	Medium	7.67E+07	3.53E+06	2.12E+06	1.19E+06	4.02E+05
	High			2.82E+06	1.58E+06	5.36E+05
Sub-Saharan Africa	Low			9.06E+05	5.07E+05	1.72E+05
	Medium	2.26E+06	1.36E+06	1.36E+06	7.61E+05	2.58E+05
	High			1.81E+06	1.01E+06	3.44E+05
Middle East & North Africa	Low			6.53E+05	3.66E+05	1.24E+05
	Medium	6.80E+07	1.63E+06	9.79E+05	5.48E+05	1.86E+05
	High			1.31E+06	7.31E+05	2.48E+05
All	Low			9.99E+06	5.60E+06	1.90E+06
	Medium	7.36E+08	2.50E+07	1.50E+07	8.40E+06	2.85E+06
	High			2.00E+07	1.12E+07	3.80E+06

^a^“Open dumped waste” was calculated using country-level MSW generation data from the  covering 2011-2017, including categories reported as “dumped”, “other”, and “uncounted.” For key countries in regions with the greatest extent of dumped waste, values were updated, using more recent literature where such data were available. ^b^“Textile waste dumped” estimated as a percentage of total openly dumped MSW, based on country-level waste composition data where available, and supplemented with a global weighted average of 3% where national statistics on textile waste were unavailable (Table 1). ^c^“Textile waste burned” (i.e. OBTW) assumes that 60% (default IPCC value), 40% (low), and 80% (high) of dumped textile waste undergoes entirely burned, based on guidance from the 2006 IPCC Guidelines for National Greenhouse Gas Inventories (IPCC, 2006). ^d^“Polyester burned” was calculated as 56% of burned textile waste, based on global polyester fiber production (57%) and adjusted for the estimated proportion of recycled polyester textiles (≈1%) (Textile Exchange, 2024). ^e^“Cotton burned” was calculated as 19% of burned textile waste, based on global cotton fiber production (20%) and adjusted for the estimated proportion of recycled cotton textiles (1%) (Textile Exchange, 2024)

### Textile waste burning estimates

We estimate that around 25 Mt of textile waste is dumped globally each year, with India (8 Mt), China (2 Mt), and Indonesia (1.7 Mt) leading the top three countries in terms of volume. Applying burning fractions of 40%, 60%, and 80% (IPCC 2006; Gómez-Sanabria et al. 2022), we calculate that 10–20 Mt (medium 15 Mt) of textile waste is burned annually in uncontrolled, open conditions, distinct from controlled incineration. As Table 5 illustrates, the largest regional contributions come from South Asia (3.8–7.7 Mt, medium 5.8 Mt) and East Asia and Pacific (2.1–4.3 Mt, medium 3.2 Mt).

**Fig. 3:**
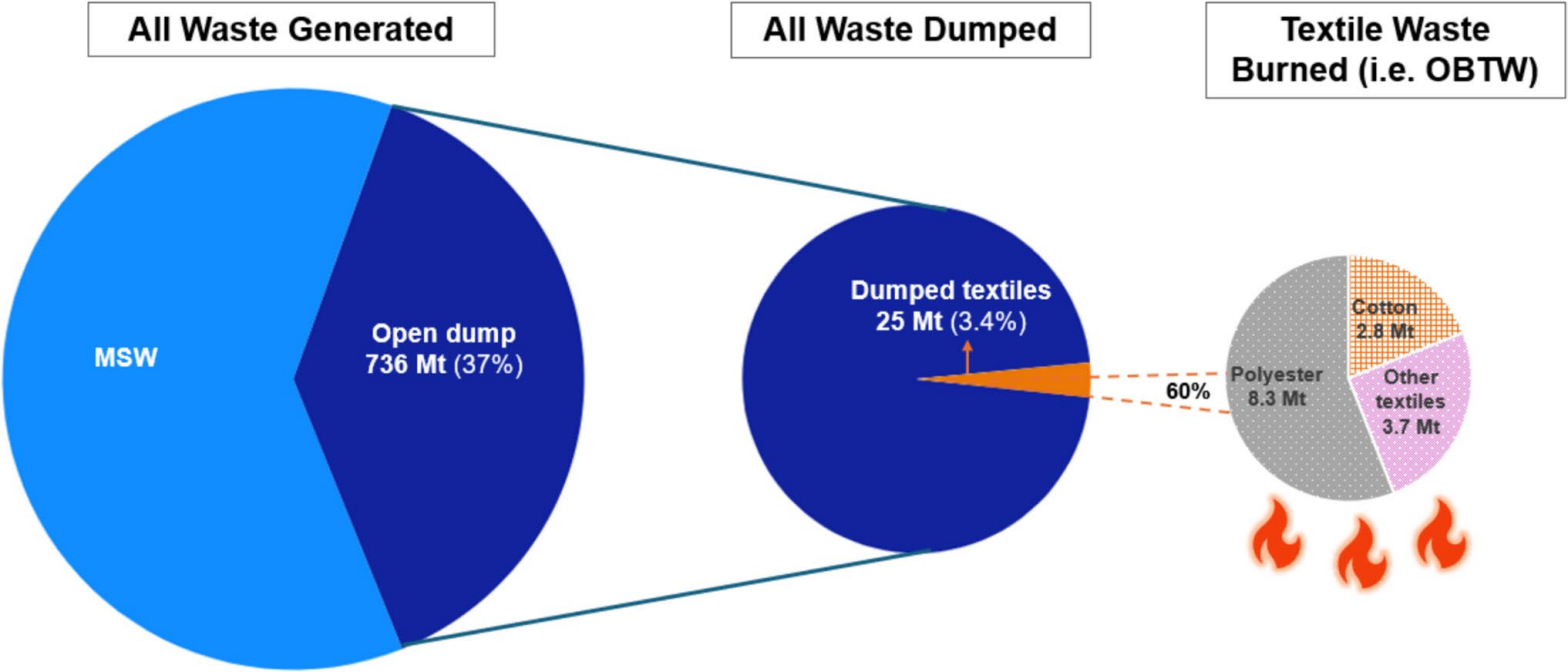
Waste stream flow shows polyester and cotton volumes in OBW. Of all MSW generated, 37% is openly dumped. Within this, 3.4% represents dumped textiles, while the right pie chart separately shows the composition of OBTW (textile waste that is burned in open environments). Values are based on medium estimates; the full range of burned textile shares by region is provided in Table 5

Given the lack of specific data on the composition of dumped waste, the textile fraction in MSW was applied as an assumption to derive these estimates. Fiber-type breakdowns were based on global production shares (Textile Exchange 2024), resulting in 8.3 Mt of polyester (range 5.5–11 Mt) and 2.8 Mt of cotton (range 1.8–3.7 Mt) burned. The remainder (25%) consists of other synthetics (e.g., viscose, polyamide, blends, plant fibers other than cotton, and animal fibers such as wool (Fig. 3).

### Gaseous emissions generated from polyester and cotton OBTW

Our estimations indicate that although textiles represent only about 3% of global MSW, the emissions resulting from open burning, especially polyester, pose significant health and environmental risks, particularly for communities living near open-burning sites. Polyester fiber, a synthetic polymer known as polyethylene terephthalate (PET), produces emissions comparable to those from plastic (e.g. PET, PE) combustion, including GHGs, black carbon, organic pollutants such as PAHs and c-PAHs (carcinogenic PAHs), dioxins, toxic metals (Pathak et al. 2024).

#### Polyester emissions

When comparing methods of burning, polyester textiles contribute significantly to atmospheric pollution, particularly through barrel burning. By using emission factors from Cheng et al. (2020), we calculated that polyester textile burned in barrels emits 15.1 Mt (range 10–20 Mt) of fossil CO_2_ annually, significantly more than pile burning (8.3 Mt, range 5.5–11 Mt of CO_2_) (Table 6). This pattern holds true for other pollutants, such as CO, NOx, and SO_2_, where emissions from barrel burning are consistently higher than those from pile-up burning. According to Cheng et al. (2020), these emissions might be influenced by the combustion process itself, which can be categorized into three phases: flaming, transition, and smoldering. The modified combustion efficiency (MCE) is used to differentiate between these phases: an MCE above 0.9 indicates flaming, while below 0.9 suggests smoldering. Barrel burning often involves restricted and uneven oxygen supply, creating alternating smoldering and localized high-temperature combustion zones, whereas pile burning allows greater air exchange and shorter burning duration. These combined combustion conditions can increase total pollutant release per mass burned compared with open pile burning, although the dominant formation mechanisms differ among pollutant types.

When comparing barrel emissions with those derived from the ecoinvent v3.5 life-cycle inventory database (Wernet et al. 2016), which considers uncontrolled burning conditions even greater differences are observed. For instance, while the CO_2_ emission factor for plastic in barrel burning is 1.8 kg/kg, the ecoinvent emission factor is 2.92 kg/kg. This discrepancy leads to a significant difference in total fossil CO_2_ emissions, with the ecoinvent-based method resulting in 24.5 Mt (range 16–32 Mt) of CO_2_ emissions—a 38% increase compared to the barrel burning method. Similarly, Wang et al.’s (2023) study on PE (plastic bags) showed a comparable CO_2_ emission factor of 2.93 kg/kg, totaling 24.6 Mt of carbon dioxide. To further understand these discrepancies, we calculated the theoretical CO_2_ emissions for PET combustion based on the molecular weight of its repeat unit. PET has a repeat unit molecular weight of 192.17 g/mol (C_10_H_8_O_4_), yielding a theoretical CO_2_ emission of approximately 2.29 kg CO_2_ per kilogram of PET burned. This calculation, however, is based on a single repeat unit and assumes complete combustion, which does not fully capture real-world burning conditions in which PET’s polymer structure varies in chain length, and combustion may be incomplete, and thus impacting the oxidation factor.

#### Calculation of theoretical CO_2_ emissions from PET

PET’s repeat unit formula and molar mass—C_10_H_8_O_4_ (192.17 g/mol)

Molar mass of CO_2_—44.01 g/mol, where each mole of PET produces 10 mol of CO_2_.

Moles of PET per kg (1000 g):

$$\frac{1000\mathrm{g}}{192.17\text{ g}/\mathrm{mole}}=5.2\text{ moles of PET}$$

CO_2_ produced per kg of PET:

$$5.2\text{ moles of PET}*10\text{ moles of }{\mathrm{CO}}_{2}\text{ per mole of PET}*44.01\text{ g}/\text{mol }{\mathrm{CO}}_{2}=2.29\text{ kg of }{\mathrm{CO}}_{2}$$

In essence, a higher emission factor for PET in uncontrolled burning likely incorporates assumptions about incomplete combustion, thus additional oxidation of byproducts, and adjustments for variability in burning efficiency. Incomplete combustion means not all carbon atoms in PET are fully converted to CO_2_. Instead, some carbon is released as carbon monoxide (CO), particulate matter (e.g., black carbon or soot), or even unburned hydrocarbons (IPCC 2006). Thus, this factor may account for the release of these other carbon-containing compounds, which results in a lower CO_2_ yield per unit of PET burned, but it could also assume that CO and hydrocarbons eventually oxidize to CO_2_ in the atmosphere, effectively increasing the CO_2_-equivalent emissions. During the oxidation process, some other pollutants like volatile organic compounds (VOCs), PMs, PAHs, and dioxins are released causing negative short-term and long-term health effects (Tomsej et al. 2018).

There are also differences in emissions of other gases, such as SO_2_, where lower values (672 t, range 448–896 t) are based on Wang et al.’s (2023) emission factors, compared to 4198 t (range 2799–5597 t) from barrel burning. All these discrepancies emphasize the need for further research to understand how geographical, environmental, and physical factors may influence emissions. Overall, CO_2_ emissions are the biggest share of total emissions, and South Asia alone is estimated to contribute around 37% of these emissions among other regions (see Figure S2 for country-specific CO_2_ emissions). A city-level example illustrates the air-quality implications of these emissions: in Mumbai—one of India’s largest urban centers—OBW accounts for about 20% of ambient air pollution (Lal et al. 2016; Ramadan et al. 2022), demonstrating how high regional OBTW levels could manifest as substantial local pollution burdens.

#### Cotton emissions

Cotton, primarily composed of cellulose, behaves similarly to paper during combustion. As a biogenic material, the carbon released during burning is part of the natural carbon cycle and is therefore considered carbon-neutral in most life cycle assessments (LCAs) (Thylmann & Kießer 2024). On the global scale, however, polyester burning contributes 3–5 times more carbon emissions than cotton (which also has a high carbon content), owing to its higher production share and fossil-based origin. For cotton, CO_2_ emissions from OBTW are estimated between 2.8 and 4.2 Mt, with the highest recorded by Wang et al. (2023) and Cheng et al. (2020) using the barrel method, whereas pile burning results in the lowest CO_2_ emissions (Table 6).

Burning cotton may still generate other pollutants. Using paper combustion as a proxy, emission factors indicate that this process can release relatively higher amounts of NOx and SO_2_ compared to polyester (Cheng et al. 2020; Wang et al. 2023). These pollutants are common to the combustion of many biomass-based materials and may influence local air quality and respiratory health. Therefore, while cotton’s GHG-related global climate impact can be lower, localized pollutant generation remains an important consideration for all materials when burned under uncontrolled conditions.

Despite the lower waste volumes, CO emissions from cotton are also not significantly lower than those from polyester. This may be due to the material’s combustion properties, as suggested by Cheng et al. (2020), where factors like moisture content and density can influence emissions. Total gaseous emissions (CO_2_, CO, SO_2_, NOx) from cotton average between 2.9 and 4.3 Mt, while polyester emissions range from 8.5 to 24.8 Mt, indicating greater environmental impact of polyester burning.

Regarding CH_4_ emissions, there is limited data for both plastic and paper emission factors. Ecoinvent v3.5 database reports an emission factor of 5.9 g/kg for both, categorized as process-specific emissions from open burning. The IPCC reports a default emission factor of 6.5 g/kg MSW wet weight for open burning of waste (IPCC 2006). As polyester is derived from petroleum, its CH_4_ content may differ, making this an area that requires further research. Based on ecoinvent factors, CH_4_ emissions from polyester and cotton would be 49,534 t (range 33,023–66,046 t) and 16,806 t (range 11,204–22,408 t), respectively.

#### Comparative LCA of polyester and cotton in textile open burning

Overall, open burning of textile waste, including polyester and cotton, reveals clear differences in their carbon dioxide emissions. Cotton, being a biomass-based material, mostly contains biogenic carbon that is released as CO_2_ during burning. Since biogenic CO_2_ is part of the natural carbon cycle, it is considered carbon-neutral and assigned a global warming potential (GWP) of zero in bio-product life cycle assessments (LCAs) (Saade et al. 2020). Regarding raw material production, the largest contributors to cotton’s GWP come from fertilizer production (27%) and nitrous oxide emissions (35%) during cultivation. Despite this, cotton’s overall GWP is negative (−112 kg CO_2_ eq. per 1000 kg of fiber), as it absorbs more carbon than it emits during production (Cotton Incorporated 2017).

In contrast, polyester, derived from fossil fuels, releases fossil CO_2_ during combustion, contributing to long-term atmospheric carbon accumulation. The carbon footprint of raw polyester textile production is 119.59 kg CO_2_/100 kg (Tekin et al. 2024). According to the IPCC guidelines, CO_2_ emissions resulting from the combustion of fossil-based materials, such as plastics and synthetic textiles, are considered net emissions and should be included in national CO_2_ emission estimates. This distinction highlights the greater climate impact of polyester waste burning compared to natural fibers like cotton.

### PMs, PAHs, and THMs from polyester and cotton OBTW

Open burning is a significant source of hazardous pollutants, including black carbon (BC), which has a much higher global warming potential than methane. It is reported that BC from open burning produces a CO_2_ equivalent (CO_2_eq) that is four times larger than methane emissions from waste decomposition (Reyna-Bensusan et al. 2019). However, BC emissions are often underestimated and excluded from IPCC methodologies, making accurate quantification challenging (Ramadan et al. 2023). As a component of PM_2.5_ and PM_10_, black carbon contributes significantly to air pollution and poses serious health risks. In China PM_10_ emissions from domestic waste burning account for 22% of the country’s total anthropogenic PM_10_ emissions, with plastics contributing 90% of the black carbon released (Pathak et al. 2024).

Our analysis indicates that polyester generates higher PM_2.5_ and PM_10_ emissions compared to cotton across all methods. The highest emissions were reported by Wang et al. (2023), with polyester burning releasing 285,452 (range 190,301–380,602) tons of PM_2.5_ and 306,861 (range 204,574–409,148) tons of PM_10_, compared to cotton (37,914, range 25,276–50,552) tons of PM_2.5_ and (38,227, range 25,485–50,970) tons of PM_10_. In contrast, ecoinvent’s emission factors show much lower values, with 8270 (range 5513–11,026) tons of PM_2.5_ and 3182 (range 2121–4243) tons of PM_10_ for polyester and 2962 (range 1975–3950) tons of PM_2.5_ and 1145 (range 763–1527) tons of PM_10_ for cotton. These discrepancies arise from varying emission factors, emphasizing critical research gaps. For instance, PM_10_ emission factors vary widely between ecoinvent v3.5 database (0.379 g/kg for PE and 0.402 g/kg for paper), Hoffer et al. (2020) (11 g/kg for PET and 2.2 g/kg for paper), and Wang et al. (36.5 g/kg for PE and 13.4 g/kg for paper), highlighting the urgent need for further investigation.

Regarding PAH emissions, available data is also limited. Ecoinvent v3.5 database applies the same emission factor for both PE and paper (0.344 g/kg) in process-specific PAH emissions from open burning, leading to 2,888 (range 1,925–3,851) tons of PAH emissions from polyester and 980 (range 653–1,307) tons from cotton. Hoffer et al.’s (2020) method produced lower results for polyester—269 (range 179—358) tons of PAH emissions, but significantly lower emissions for cotton—3 (range 2–5) tons of PAHs. Tomsej et al. (2018) found that PAH emissions from polyester co-combustion are particularly high, especially for carcinogenic PAHs, which pose substantial health risks.

Globally, OBW contributes to 61% of total PAH emissions, 29% of PM2.5, and 10% of mercury (Hg, or THMs), worsening regional haze and environmental quality (Lal et al. 2016; Wiedinmyer et al. 2014). In many developing countries, such as China, household garbage is often burned directly outdoors in piles or barrels, particularly in rural areas, where this rudimentary practice is sometimes used for heating (Cheng et al. 2020; Ramadan et al. 2023). A study by Pansuk et al. (2018) indicated that over 50% of domestic waste in rural Thai communities is burned, with plastics comprising at least 30% of the waste. The proximity of burning sites to densely populated areas in these regions heightens health risks for local residents (Ramadan et al. 2023). A study by Cheng et al. (2020) on China’s emissions from OBW found that hazardous substances such as PM_2.5_ made up 8.7% of emissions, and THMs averaged 3.8%.

PM air pollution is a major contributor to the global burden of disease, and the most significant environmental factor contributing to morbidity and mortality worldwide (Kodros et al. 2016). In 2019, long-term exposure to PM_2.5_ was linked to approximately 4.14 million deaths globally, accounting for 62% of all air pollution-related deaths (State of Global Air 2019). Over the past decade, deaths attributed to PM_2.5_ increased by around 23%, with Asia and Africa bearing the highest burden (State of Global Air 2019). Detailed emissions data for PM_10_ and PAHs on a regional basis can be found in Table S1. Our estimates of toxic heavy metal emission generation capacity show that arsenic (As) and zinc (Zn) tend to be released at higher rates from plastics than from cellulose-based materials, whereas lead (Pb) exhibits a slightly higher emission rate from cellulose-based materials (Table 6).

### Carcinogenic PAHs

The health risks associated with plastic burning are heightened by the specific type of plastic, as some release carcinogenic or toxic pollutants (Pathak et al. 2024). Recent studies have identified over 16,000 substances associated with plastics and plastic products, more than 4200 of which are considered hazardous due to their persistence, bioaccumulation, mobility, and toxicity (Wiesinger et al. 2024). This chemical complexity intensifies concerns over combustion byproducts, especially when waste is burned under uncontrolled conditions. Among the most hazardous combustion products are carcinogenic PAHs (cPAHs), a class of compounds known to be mutagenic and carcinogenic. Of the 16 US Environmental Protection Agency (EPA) priority PAHs, the most carcinogenic include five-ring compounds such as benzo(a)pyrene (BaP), dibenzo(a,h)anthracene, and benzofluoranthenes, where BaP is frequently used as a marker for cPAHs due to its high toxicity (Hoffer et al. 2020; Zelinkova & Wenzl 2015). Using toxic equivalency factors, where BaP is assigned a reference value of 1, Hoffer et al. (2020) estimated the PAH emission factor from PET combustion—expressed in BaP toxicity equivalents—at 2.2 mg/kg. This is nearly 14 times higher than that of paper (0.16 mg/kg), corresponding to total emissions of approximately 18.5 (range 12.3–24.6) tons for PET versus 0.5 (range 0.3–0.6) tons for cellulose-based materials (with paper used as a proxy for cotton). Although plastics can produce substantially higher PAH and BaP-equivalent emissions than cellulosic materials under low-temperature or oxygen-limited burning conditions (Hoffer et al. 2020; Lemieux et al. 2004), PAH formation is highly sensitive to combustion parameters such as temperature, oxygen availability, and waste composition. Current data remains limited, particularly for textile waste. Further research under realistic open-burning conditions is needed to better characterize the specific PAH and BaP-equivalent emissions from these materials.

**Fig. 4:**
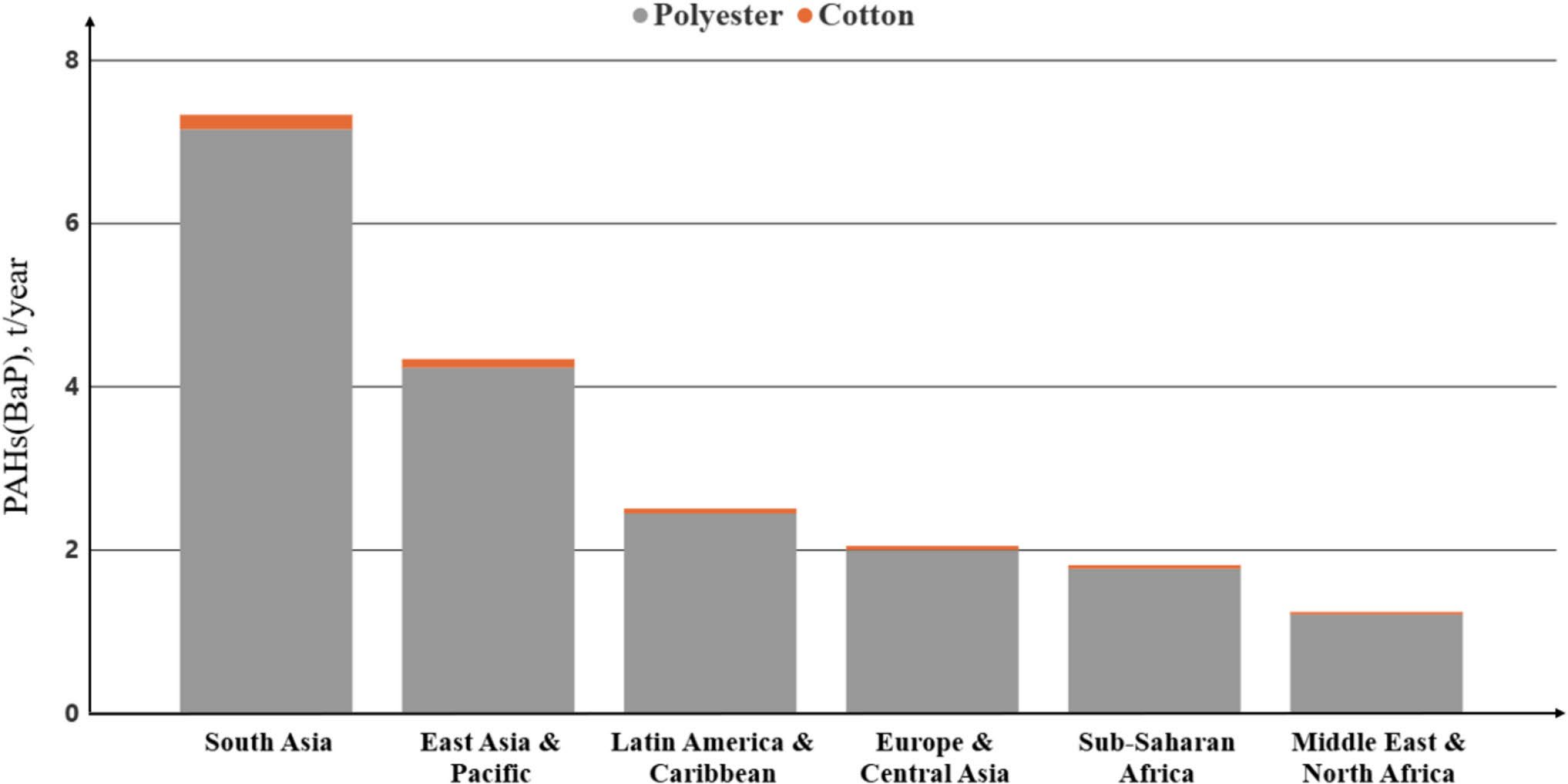
Emissions of cPAHs from polyester and cotton by regions, where PAHs (BaP) is the total PAHs expressed in BaP Toxicity Equivalent

Although cPAHs emissions might seem negligible compared to other PAH sources, they represent significant health risks due to their carcinogenic nature, particularly in areas where residential waste burning is used for heating, often indoors. Our calculations show that Asian countries dominate cPAHs emissions from polyester, contributing nearly half of the total, further exacerbating public health concerns in densely populated regions where waste burning is prevalent (Fig. 4). Detailed total emissions from cotton and polyester burning using different methods are shown in Table 6. The low- and high-scenario ranges are presented in Tables S2, S3.

**Table 6:** Emissions generated during polyester and cotton OBTW

OBTW	Method applied	Emissions generated, t/year												
		Fossil CO_2_^a^	Biogenic CO_2_^b^	CO	NOx	SO_2_	CH_4_	PM_2.5_	PM_10_	PAHs	PAHs (BaP)^c^	THMs^d^ (As)	THMs (Pb)	THMs (Zn)
Polyester	Barrel (Cheng et al. 2020)	1.51E+07		3.64E+05	2.60E+04	4.20E+03		1.09E+05				1.60E+04	1.01E+04	3.19E+03
Polyester	Pile (Cheng et al. 2020)	8.40E+06		1.70E+05	9.24E+03	2.52E+03		3.78E+04				1.09E+04	9.24E+03	1.43E+03
Polyester	Field (ecoinvent v3.5 database)	2.45E+07		3.24E+05	2.01E+04	1.54E+03	4.95E+04	8.27E+03	3.18E+03	2.89E+03		1.30E-01	1.60E+00	2.13E+01
Polyester	Winter stove (Hoffer et al. 2020)								9.24E+04	2.69E+02	1.85E+01			
Polyester	Lab (Wang et al. 2023)	2.46E+07		1.88E+05	1.26E+04	6.72E+02		2.85E+05	3.07E+05					
Cotton	Barrel (Cheng et al. 2020)		3.99E+06	1.90E+05	9.40E+03	8.55E+02		2.99E+04				2.85E+03	5.41E+03	8.26E+02
Cotton	Pile (Cheng et al. 2020)		2.85E+06	9.71E+04	6.55E+03	5.70E+02		1.71E+04				3.42E+03	5.70E+03	3.42E+02
Cotton	Field (ecoinvent v3.5 database)		3.99E+06	1.10E+05	1.53E+04	1.73E+03	1.68E+04	2.96E+03	1.15E+03	9.80E+02		4.98E-02	1.92E+00	2.65E+00
Cotton	Winter stove (Hoffer et al. 2020)								6.27E+03	3.42E+00	4.56E-01			
Cotton	Lab (Wang et al. 2023)		4.27E+06	1.28E+05	3.25E+03	1.62E+03		3.79E+04	3.82E+04					

^a^Burning fossil–based products like plastics, which are made from oil, releases fossil carbon into the atmosphere. ^b^Biogenic carbon emissions come from biological sources such as plants, trees, and soil and are part of the natural carbon cycle. ^c^Emissions of total polycyclic aromatic hydrocarbons (PAHs) expressed in benzo(a)pyrene (BaP) toxicity equivalent. ^d^Toxic heavy metals (THMs) substances contained in PM_2.5_ emissions. Note: Emissions were calculated using medium-scenario OBTW values from Table 5 and pollutant emission factors listed in Table 3

### Sensitivity analysis: drivers of CO_2_ uncertainty

**Fig. 5:**
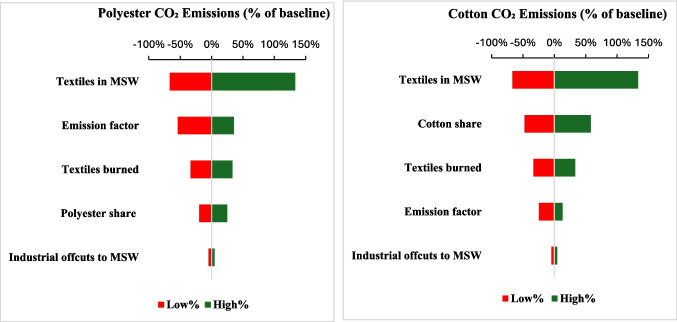
Tornado sensitivity plots for polyester and cotton CO_2_ emissions from open burning of dumped textiles. Bars show percent change relative to baseline when setting each input at low and high values (one at a time), holding others at baseline

To assess robustness, we conducted a one-at-a-time sensitivity analysis varying five inputs between literature-based low/high values while holding others at baseline. Figure 5 presents tornado plots for polyester and cotton, showing percent deviation from baseline CO_2_ emissions.

Across both fibers, textiles in MSW are the dominant drivers (about −67 to +133%). For polyester, the CO_2_ emission factor ranks second (≈−54 to +36%), followed by the share burned (≈±33%). For cotton, the cotton share of textile waste exerts a larger influence (≈−48 to +58%) than the emission factor (≈−24 to +13%). Industrial offcuts entering MSW contribute comparatively little (≈±5%). These results indicate that waste composition audits (textile fraction) and fiber-specific emission factors (especially for synthetics) are the most impactful data needed for reducing inventory uncertainty.

## Research and data gaps

This study is based on secondary datasets, primarily the World Bank’s *What a Waste* database, which remains the most comprehensive global source of municipal waste data. However, it covers the period 2011–2017, and no consolidated updates are available since the COVID-19 global pandemic. The data on open dumping represents recorded instances but may not capture all countries where OBW persists informally despite legal prohibitions. In rural areas, particularly in some Eastern European countries within the EU, open burning is illegal but may persist unofficially (Hoffer et al. 2020). However, some EU countries, despite having stringent waste management legislation, still have reported instances of open dumping. In certain rural areas of EU countries, OBW may still occur, as seen in Hungary, Poland, and Romania, as reported by Hoffer et al. (2020). Nevertheless, such incidents are negligible (accounting for less than 1%) when compared to the widespread practices observed in many Asian and African countries. Overall, the findings highlight the need for updated, region-specific datasets and direct field measurements of open-burning activity to refine future emission inventories.

### Textile waste composition

Assessing the textile fraction within open dumped waste and distinguishing between natural and synthetic fibers presents further complications. According to the recent report by Fashion for Good, cotton accounts for 47% and polyester for 33% of the apparel waste stream in US landfills (FFG 2024). However, this contrasts with global fiber production, where polyester (57%) outweighs cotton (20%) (Textile Exchange 2024). The variation likely stems from non-apparel applications of polyester, which may not enter municipal waste streams in the same proportions. Given the wide-ranging estimates of polyester used for non-apparel purposes (5–50%) (FFG 2024), further research is required to refine our understanding of global polyester fiber flows. Additionally, textile waste management varies significantly by region. While high-income countries often have structured disposal and recycling systems, low- and middle-income countries, which frequently receive secondhand textiles, lack comprehensive tracking mechanisms.

### Secondhand apparel waste in importing countries

For major importers of secondhand textiles, the lack of comprehensive waste audits makes it difficult to track the fate of incoming bales, quantify the resulting textile waste, or attribute disposal pathways. Addressing these data gaps is critical for accurately estimating emissions from open burning and decomposition, thereby supporting better waste management policies.

#### Global trade context

UN Comtrade data indicate that in 2021, the European Union (30%), China (16%), and the USA (15%) were the leading exporters of discarded clothes, while Asia (28%; predominantly Pakistan), Africa (19%; notably Ghana and Kenya), and Latin America (16%; mainly Chile and Guatemala) were the principal importers (UNECE 2024). These flows underline why many waste burdens materialize in importing rather than exporting regions.

#### Ghana

Ghana is one of the major importers of secondhand textiles and, in 2021, was the world’s 12th largest importer, accounting for 2% of global imports (Ellen Macarthur Foundation 2024). The Kantamanto Market in Accra, one of the world’s largest secondhand clothing hubs, processes approximately 70,000 tons of used textiles annually (Ellen Macarthur Foundation 2024). Around 40% of these textiles become waste, 70% of which is abandoned in the environment, while only 30% is collected for landfill disposal (Ellen Macarthur Foundation 2024; The Or Foundation 2022). However, no data on open dumping or burning of this waste was recorded in the World Bank dataset we used for this study.

#### Chile

Based on our calculations, Chile generates approximately 6.5 million tons of MSW annually, with an estimated 900,000 tons dumped illegally and with the textile dumped waste of 27,000 tons. Meanwhile, the country receives millions of tons of secondhand apparel annually, by some reports up to 46 million tons (Bartlett 2024), mainly through the duty-free port of Iquique. Unsold clothing, often composed of synthetic materials, is discarded in illegal dumpsites, including those in the Atacama Desert (Bartlettc 2024). This accumulation highlights a potential discrepancy between imported textile waste and recorded MSW figures, suggesting an underestimation of emissions from open burning and textile decomposition.

#### Pakistan

Pakistan is a major importer and sorter of secondhand apparel, with textile waste becoming a major environmental concern, with millions of tons of discarded fabrics and garments ending up in landfills annually (Hussain et al. 2025). A 2025 NTU–Reverse Resources mapping estimated only pre-consumer textile waste is about 887,000 tons annually, while local post-consumer textile waste is 270,000 tons and imported second hand around 809,000 tons (Hussain et al. 2025).

### Industrial leakage caveat

Our OBTW estimates are MSW-based, but there is documented evidence that pre-consumer offcuts from textile manufacturers (industrial waste) can flow into municipal disposal or be openly burned. Case studies report tailoring offcuts in India (Udaipur) being openly dumped or sent to landfill (Jaymala & Sudha 2020), and in Nigeria (Enugu) being indiscriminately disposed in dumps and drainage canals—both indicating entry into public waste pathways (Silas-Ufelle 2025). National policy work in Bangladesh on textile “jhut” (cutting waste) also lists landfill and burning/incineration among disposal routes (Hoque et al. 2024) underscoring plausible open-burning pathways even though no country publishes a measured national share of industrial textile waste entering MSW. The composition of pre-consumer waste varies by country. For Pakistan, cotton-based materials dominate (68%) and synthetics are 26% (Hussain et al. 2025). In Vietnam, recent ecosystem mapping found 35% polyester-rich, 20% cotton-rich, and 45% blended composition of pre-consumer waste (Phan et al. 2024). In Sri Lanka, most pre-consumer waste is polyester, nylon, or blends, with synthetics predominant in factory offcuts (Sulochani et al. 2024). In China, chemical fibers, including synthetics and man-made cellulosic fibers, account for more than 85% of total fiber processing and reached nearly 65 Mt in 2023 (ChinaDaily 2023). While production shares are not a direct measure of waste composition, they indicate a strong likelihood that pre-consumer offcuts are predominantly synthetic in China. Accordingly, any industrial contribution to MSW-linked OBTW likely represents a potential but currently unqualified undercount in our MSW-based estimates.

### Emission factors and environmental impact

A critical data gap exists in quantifying emissions from the open burning of synthetic and natural textile waste. Currently, there are no standardized emission factors for burning textiles, and these emissions are generally not well captured in product LCAs. The lack of precise emission factors makes it difficult to measure the true environmental impact of textile waste disposal practices. One of the core challenges in estimating emissions lies in the complex and heterogeneous composition of apparel textiles. Most garments today are made from fiber blends, with cotton-polyester composites among the most common (Xia et al. 2024), complicating combustion chemistry and emission profiles. The varying proportions of synthetic and natural fibers in these blends make it difficult to model combustion behavior and emissions with any uniform standard.

In addition to the emissions studied in this analysis, open textile waste burning releases a wide range of hazardous air pollutants, including carcinogens such as dioxins and benzene. However, emission factors for these pollutants remain highly uncertain and severely underreported. Major databases like ecoinvent v3.5database assign the same emission values to plastic and paper, despite their differing combustion behaviors. Thus, there is a critical need to establish textile-specific emission profiles that can more accurately capture the release of toxic compounds during open-burning events.

Recent observations in Ghana underscore the urgency of this issue. Greenpeace researchers found that textile waste from Accra’s Kantamanto Market is frequently burned as fuel in nearby informal settlements. Air samples collected in these areas revealed elevated levels of benzene exceeding European safety limits, thereby exposing workers and local residents to hazardous emissions (Greenpeace Africa 2024). To improve the accuracy of emissions estimation and enable more effective mitigation strategies, future research must develop context-specific emission factors that account not only for the physical and chemical properties of blended textiles but also for geographical, environmental, and combustion variables. Without such refinements, the contribution of textile waste to global emissions, and to public health risks, will remain underestimated, most likely limiting the effectiveness of policies aimed at mitigating its environmental impact.

### Carbon sequestration in textile waste management

Open burning and dumping textile waste overlook a critical climate consideration such as the loss of biogenic carbon stored in natural fibers like cotton. These textiles derive carbon from atmospheric CO_2_ via photosynthesis, meaning their disposal contributes not only to pollution but also forfeits potential carbon sequestration (Clauser et al. 2025). Emerging research highlights the controlled burial of biomass as a viable, low-cost carbon dioxide removal (CDR) strategy (Clauser et al. 2025; Zeng et al. 2013). However, the role of textiles in such frameworks remains largely unexamined. There is currently no standardized method for evaluating the carbon removal potential of buried textile waste, nor any integration of textile waste pathways into carbon markets despite growing demand for durable CDR credits (Chen et al. 2024). This gap is especially relevant for low- and middle-income countries, where infrastructure for recycling or incineration is limited, and open burning remains prevalent. Future research is needed to assess the feasibility and climate benefits for textile burial as an alternative end-of-life strategy, potentially turning a major environmental liability into a climate mitigation tool.

## Conclusion

Waste management has emerged as one of the most pressing challenges in today’s world, with textile waste surging in recent years due to various factors including population growth and fast fashion trends. A significant portion of this waste is either dumped or burned in landfills and residential areas, complicating management efforts. This practice not only harms the environment and human health but also exacerbates climate change through the release of greenhouse gases, particularly from synthetic materials. To address these challenges, improving textile waste management practices is essential. Options like recycling, composting, and biogas generation hold promise, alongside innovative scientific solutions that transform waste fabrics into valuable products. Implementing supportive policies and standards, collaborating with brands, and educating consumers can significantly contribute to solving this growing problem.

While this study is based on secondary datasets, uncertainty was explicitly assessed through low–medium–high-scenario modeling and one-at-a-time sensitivity analysis. These approaches quantified the influence of key parameters such as textile fractions, emission factors, and industrial leakage, thereby improving transparency and identifying where better data collection is most needed. Future research should incorporate field-based measurements of textile burning frequency, composition, and emissions to refine and validate modeled results.

The findings also have clear implications for emerging Extended Producer Responsibility (EPR) and circular-textile frameworks. Current EPR schemes mainly emphasize collection and recycling but rarely account for end-of-life pathways such as open dumping and burning, particularly in low- and middle-income countries. Integrating these uncontrolled disposal routes into global reporting and policy frameworks would strengthen accountability and encourage brands and producers to invest in design-for-recycling, traceability, and infrastructure development that prevent synthetic fibers from entering informal waste systems. Aligning emission data with circular-economy strategies can thus bridge the gap between waste governance, climate mitigation, and public health protection.

Key summary points are the following:

- This study reveals that 137 countries across six regions dispose of more than 700 Mt of dumped waste, with an estimated 15 Mt (range 10–20 Mt) of textile waste burned annually.
- Burning polyester may generate between 8.3 Mt (range 5.5–11 Mt) and 24.6 Mt (range 16.4–32.8 Mt) of fossil CO_2_ annually, with South Asia contributing 37% of these emissions. Emissions from burning polyester in barrels may produce more pollutants than open-pile burning.
- Open burning is a significant source of hazardous air pollutants, including PM_2.5_, PM_10_, PAHs, cPAHs, and THMs. Burning polyester and other synthetic fibers may release more pollutants compared to natural fibers, placing communities near open-burning sites in regions like South Asia at severe health risk.
- While the environmental impact of cotton appears to be generally lower than that of polyester, especially regarding CO_2_ emissions, cotton can emit comparable levels of CO and NOx under certain conditions, influenced by its combustion properties. Research gaps in methane, PAH, and other emissions indicate a need for further studies on the behavior of different textile materials during combustion.
- Due to limited data on textile waste composition and a lack of comprehensive emission factors for various textiles, further research is necessary to assess the environmental impact of textile burning fully, including polyester and cotton.
- There is an urgent need for enhanced waste management systems in low- and middle-income countries to reduce open burning. Further research is critical to accurately quantify emissions from black carbon and toxic and carcinogenic pollutants, mitigating environmental and health impacts.
- Despite growing interest in biomass burial as a climate mitigation strategy, the role of textile waste, particularly natural fibers, remains critically underexplored. Addressing this research gap is essential to evaluate the feasibility, carbon removal potential, and policy relevance of textile burial as a scalable alternative to open burning and dumping.

## Supplementary Information

Below is the link to the electronic supplementary material.

ESM1 (DOCX 205 KB)

## Data Availability

The primary dataset used to estimate open dumped textile waste volumes by country is publicly available from the What A Waste Global Database (Country-Level Dataset, last updated June 4, 2024): https://datacatalog.worldbank.org/search/dataset/0039597. Emission factors used in this study were obtained from previously published literature, as cited in the references. All additional calculations and estimates, including regional emissions by material and method, are provided in the manuscript and its supplementary information. No proprietary or restricted-access data were used.

## Abbreviations

CO: Carbon monoxide
CO_2_: Carbon dioxide
CH_4_: Methane
CDR: Carbon dioxide removal
EPA: Environmental Protection Agency
EPR: Extended Producer Responsibility
GHG: Greenhouse gas
GWP: Global warming potential
HIC: High-income countries
IPCC: Intergovernmental Panel on Climate Change
LCA: Life cycle assessment
LIC: Low-income countries
LMIC: Lower-middle-income countries
MSW: Municipal solid waste
MCE: Modified combustion efficiency
NOx: Nitrogen oxides
OBW: Open burning of waste
OBTW: Open burning of textile waste
PAH (BaP): Polycyclic aromatic hydrocarbon (benzo[a]pyrene)
PAHs: Polycyclic aromatic hydrocarbons
PE: Polyethylene
PET: Polyethylene terephthalate
PM_2.5_, PM_10_: Particulate matter with diameter ≤ 2.5 µm and ≤ 10 µm
SO_2_: Sulfur dioxide
THMs: Toxic heavy metals
VOCs: Volatile organic compounds
