# Supplementary material for: Environmental impact of open burning of polyester and cotton textile waste: a comparative analysis
Source: Environ Sci Pollut Res Int. 2026 Mar 8;33(10):4516–36. doi: 10.1007/s11356-026-37563-9 (PMC13053536; doi:10.1007/s11356-026-37563-9)
Supplement: Supplementary file 1 — (DOCX 205 KB) [file 11356_2026_37563_MOESM1_ESM.docx]

**Environmental Impact of Open Burning of Polyester and Cotton Textile Waste: A Comparative Analysis**

Jeyran Bayramova,*^a^* Steven Pires,*^a^*  Patricia A. Holden,*^c^* William J. Sagues,*^d^* Richard Venditti,*^b^* and Jesse S. Daystar *^a^*

Affiliations:

a: Cotton Incorporated, Sustainability, 6399 Weston Parkway, Cary, NC 27513, United States of America

b: North Carolina State University, Department of Forest Biomaterials, 2820 Faucette Dr., Raleigh, NC 27607, United States of America

c: Bren School of Environmental Science and Management, University of California, Santa Barbara, CA 93106, United States of America

d: Department of Biological & Agricultural Engineering, North Carolina State University, 3110 Faucette Dr., Raleigh, NC 27695, United States of America

Corresponding author: Jesse S. Daystar ([jsdaysta@gmail.com](mailto:jsdaysta@gmail.com) 919-678-2245)

**Supplementary Information**

All figures and tables are based on medium-scenario values, unless otherwise specified. Medium values represent the central estimate with the low-medium-high range of open dumped waste.

**Figure S1:** Total MSW generated and dumped waste volumes

**Figure S2:** CO_2_ Emissions from polyester and cotton OBTW by country

**Table S1.** PMs and PAHs emissions generated during polyester and cotton OBTW (t/year)

**Table S2.** Emissions generated during polyester and cotton OBTW – Low scenario (t/year)

**Table S3.** Emissions generated during polyester and cotton OBTW - High scenario (t/year)


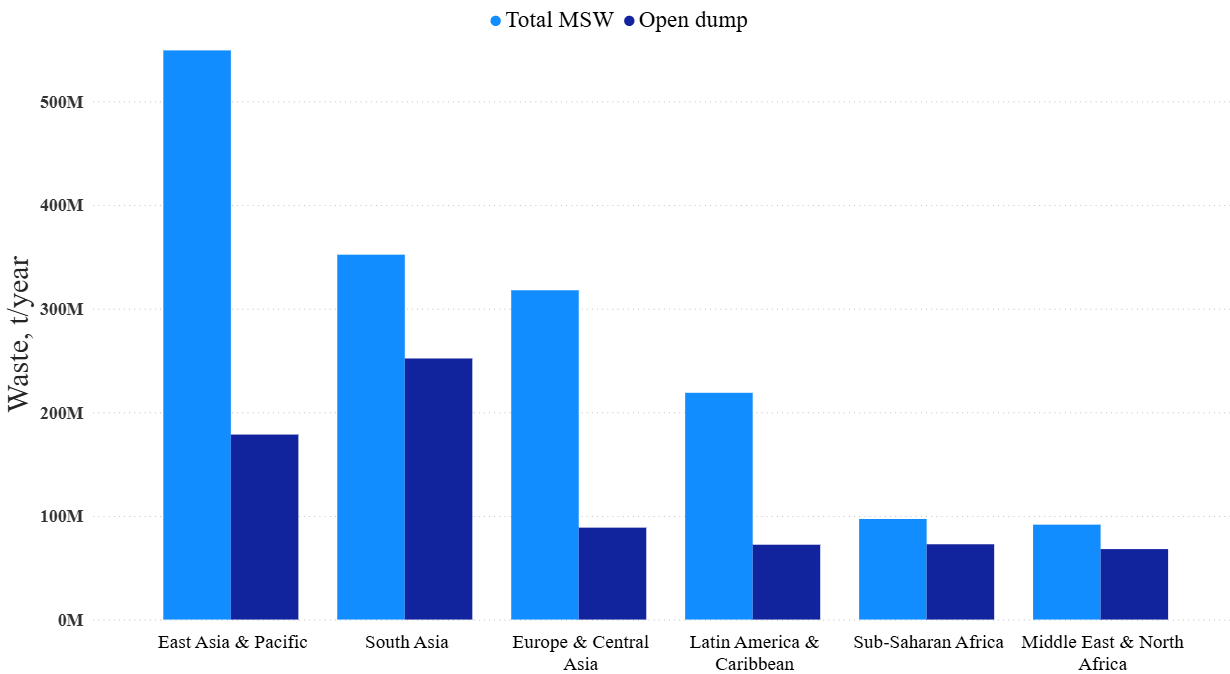


**A**

**
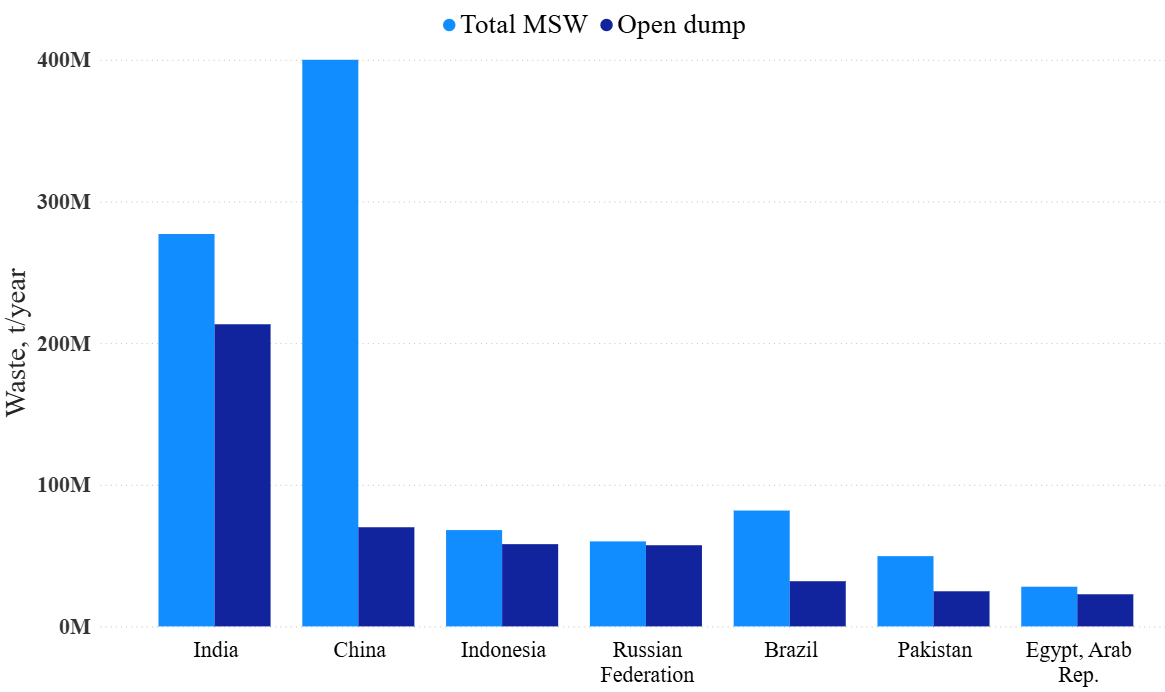
**

**B**

**Fig. S1** Total MSW generated and dumped waste volumes (*dark blue bars represent volumes of dumped waste, while light blue bars indicate total MSW volumes including sanitary landfills, incineration, and recycling*)
**A –** By region **B –** By countries with dumped waste volumes exceeding 10 Mt/year


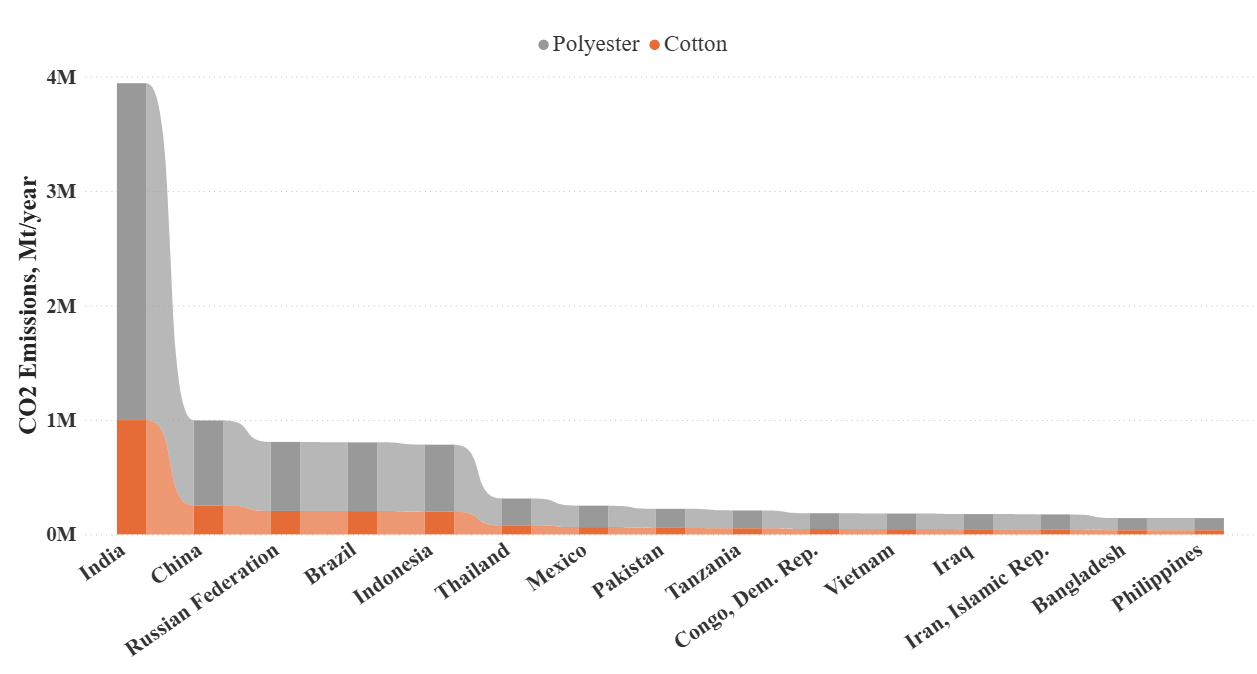
 **Fig. S2** CO_2_ emissions (Mt) from polyester (grey) and cotton (orange) OBTW by country

**Table S1** PMs and PAHs emissions generated during polyester and cotton OBTW in t/year

| **Emissions From Polyester and Cotton OBTW, t/year** | | | | | | | | |
| --- | --- | --- | --- | --- | --- | --- | --- | --- |
| **Region** | **Ecoinvent 3.5** | | | | **Hoffer et al. (2020)** | | | |
|  | **PM_10_** | | **PAHs** | | **PM_10_** | | **PAHs** | |
|  | **Polyester** | **Cotton** | **Polyester** | **Cotton** | **Polyester** | **Cotton** | **Polyester** | **Cotton** |
| South Asia | 1,232 | 443 | 1,118 | 379 | 35,743 | 2,425 | 104 | 1.3 |
| East Asia & Pacific | 693 | 249 | 629 | 213 | 20,110 | 1,365 | 59 | 0.7 |
| Europe & Central Asia | 312 | 112 | 283 | 96 | 9,059 | 615 | 26 | 0.3 |
| Sub-Saharan Africa | 288 | 104 | 262 | 89 | 8,370 | 568 | 24 | 0.3 |
| Latin America & Caribbean | 449 | 162 | 408 | 138 | 13,037 | 885 | 38 | 0.5 |
| Middle East & North Africa | 208 | 75 | 189 | 64 | 6,031 | 409 | 18 | 0.2 |
| **Total** | **3,182** | **1,145** | **2,888** | **980** | **92,352** | **6,267** | **269** | **3.42** |

**Table S2** Emissions generated during polyester and cotton OBTW (Low scenario)

| **OBTW** | **Method applied** | **Emissions generated, t/year** | | | | | | | | | | | | |
| --- | --- | --- | --- | --- | --- | --- | --- | --- | --- | --- | --- | --- | --- | --- |
|  |  | **Fossil**  **CO_2_** | **Biogenic**  **CO_2_** | **CO** | **NOx** | **SO_2_** | **CH_4_** | **PM_2.5_** | **PM_10_** | **PAHs** | **PAHs**  **(BaP)** | **THMs**  **(As)** | **THMs**  **(Pb)** | **THMs**  **(Zn)** |
| Polyester | Barrel (Cheng et al. 2020) | 1.01E+07 |  | 2.43E+05 | 1.74E+04 | 2.80E+03 |  | 7.28E+04 |  |  |  | 1.06E+04 | 6.72E+03 | 2.13E+03 |
| Polyester | Pile (Cheng et al. 2020) | 5.60E+06 |  | 1.14E+05 | 6.16E+03 | 1.68E+03 |  | 2.52E+04 |  |  |  | 7.28E+03 | 6.16E+03 | 9.52E+02 |
| Polyester | Field (Ecoinvent 3.5) | 1.63E+07 |  | 2.16E+05 | 1.34E+04 | 1.03E+03 | 3.30E+04 | 5.51E+03 | 2.12E+03 | 1.93E+03 |  | 8.68E-02 | 1.06E+00 | 1.42E+01 |
| Polyester | Winter stove (Hoffer et al. 2020) |  |  |  |  |  |  |  | 6.16E+04 | 1.79E+02 | 1.23E+01 |  |  |  |
| Polyester | Lab (Wang et al. 2023) | 1.64E+07 |  | 1.25E+05 | 8.40E+03 | 4.48E+02 |  | 1.90E+05 | 2.05E+05 |  |  |  |  |  |
| Cotton | Barrel (Cheng et al. 2020) |  | 2.66E+06 | 1.26E+05 | 6.27E+03 | 5.70E+02 |  | 1.99E+04 |  |  |  | 1.90E+03 | 3.61E+03 | 5.51E+02 |
| Cotton | Pile (Cheng et al. 2020) |  | 1.90E+06 | 6.48E+04 | 4.37E+03 | 3.80E+02 |  | 1.14E+04 |  |  |  | 2.28E+03 | 3.80E+03 | 2.28E+02 |
| Cotton | Field (Ecoinvent 3.5) |  | 2.66E+06 | 7.33E+04 | 1.02E+04 | 1.15E+03 | 1.12E+04 | 1.97E+03 | 7.63E+02 | 6.53E+02 |  | 3.32E-02 | 1.28E+00 | 1.76E+00 |
| Cotton | Winter stove (Hoffer et al. 2020) |  |  |  |  |  |  |  | 4.18E+03 | 2.28E+00 | 3.04E-01 |  |  |  |
| Cotton | Lab (Wang et al. 2023) |  | 2.84E+06 | 8.53E+04 | 2.16E+03 | 1.08E+03 |  | 2.53E+04 | 2.55E+04 |  |  |  |  |  |

**Table S3** Emissions generated during polyester and cotton OBTW (High scenario)

| **OBTW** | **Method applied** | **Emissions generated, t/year** | | | | | | | | | | | | |
| --- | --- | --- | --- | --- | --- | --- | --- | --- | --- | --- | --- | --- | --- | --- |
|  |  | **Fossil**  **CO_2_** | **Biogenic**  **CO_2_** | **CO** | **NOx** | **SO_2_** | **CH_4_** | **PM_2.5_** | **PM_10_** | **PAHs** | **PAHs**  **(BaP)** | **THMs**  **(As)** | **THMs**  **(Pb)** | **THMs**  **(Zn)** |
| Polyester | Barrel (Cheng et al. 2020) | 2.01E+07 |  | 4.86E+05 | 3.47E+04 | 5.60E+03 |  | 1.46E+05 |  |  |  | 2.13E+04 | 1.34E+04 | 4.25E+03 |
| Polyester | Pile (Cheng et al. 2020) | 1.12E+07 |  | 2.27E+05 | 1.23E+04 | 3.36E+03 |  | 5.04E+04 |  |  |  | 1.46E+04 | 1.23E+04 | 1.90E+03 |
| Polyester | Field (Ecoinvent 3.5) | 3.27E+07 |  | 4.32E+05 | 2.68E+04 | 2.06E+03 | 6.60E+04 | 1.10E+04 | 4.24E+03 | 3.85E+03 |  | 1.74E-01 | 2.13E+00 | 2.84E+01 |
| Polyester | Winter stove (Hoffer et al. 2020) |  |  |  |  |  |  |  | 1.23E+05 | 3.58E+02 | 2.46E+01 |  |  |  |
| Polyester | Lab (Wang et al. 2023) | 3.28E+07 |  | 2.51E+05 | 1.68E+04 | 8.96E+02 |  | 3.81E+05 | 4.09E+05 |  |  |  |  |  |
| Cotton | Barrel (Cheng et al. 2020) |  | 5.32E+06 | 2.53E+05 | 1.25E+04 | 1.14E+03 |  | 3.99E+04 |  |  |  | 3.80E+03 | 7.22E+03 | 1.10E+03 |
| Cotton | Pile (Cheng et al. 2020) |  | 3.80E+06 | 1.30E+05 | 8.74E+03 | 7.60E+02 |  | 2.28E+04 |  |  |  | 4.56E+03 | 7.60E+03 | 4.56E+02 |
| Cotton | Field (Ecoinvent 3.5) |  | 5.32E+06 | 1.47E+05 | 2.04E+04 | 2.30E+03 | 2.24E+04 | 3.95E+03 | 1.53E+03 | 1.31E+03 |  | 6.65E-02 | 2.56E+00 | 3.53E+00 |
| Cotton | Winter stove (Hoffer et al. 2020) |  |  |  |  |  |  |  | 8.36E+03 | 4.56E+00 | 6.08E-01 |  |  |  |
| Cotton | Lab (Wang et al. 2023) |  | 5.69E+06 | 1.71E+05 | 4.33E+03 | 2.16E+03 |  | 5.06E+04 | 5.10E+04 |  |  |  |  |  |
